# Quality Assurance Framework for the Design, Collection and Curation of Standard Data Products From a Distributed Ecology Network

**DOI:** 10.1002/ece3.74000

**Published:** 2026-08-10

**Authors:** Yvonne M. Buckley, Alain Finn, Aoife Molloy, Anna Mária Csergő, Elizabeth Crone, Annabel L. Smith, Bret D. Elderd, Caroline McKeon, Deborah Roach, Dylan Z. Childs, Glenda M. Wardle, María B. García, Maude Baudraz, Rachel M. Penczykowski, Roberto Salguero‐Gómez, Ruth Kelly, Satu Ramula, Anna Liisa Laine, Simone Blomberg, Johan Ehrlen, Jesús Villellas, Aveliina Helm, Christiane Roscher, Krista Raveala, Liv Norunn Hamre, Lotte Korell, Francis Q. Brearley, Siri Lie Olsen, Astrid Wingler, Joslin L. Moore, Ayco J. M. Tack, Anna Bucharova, Olav Skarpaas, Judit Bodis, Aldo Compagnoni, Sergi Munne‐Bosch, Zuzana Munzbergova, Joachim Töpper, Peter Vesk, Melanie Morales, Lizzie Wandrag, Marjo Saastamoinen, Martin Andrzejak, Pil Rasmussen, Christina Caruso, Richard Duncan, John Dwyer, Matthew Coghill, Ronny Groenteman, Meelis Pärtel, William K. Petry, Michele Lonati, Javier Puy, Jane Catford, Trevor H. Drees, Christoph Rosche, Katriona Shea, Simone Ravetto Enri, Jennifer R. Gremer, Balázs Deák, Lauchlan Fraser, Lauri Laanisto, Orsolya Valkó, Robert Drew Sieg, Benedicte Bachelot, Jennifer Firn

**Affiliations:** ^1^ Co‐Centre for Climate + Biodiversity + Water, School of Natural Sciences, Trinity College Dublin Dublin 2 Ireland; ^2^ Department of Biology Maynooth University Maynooth Co. Kildare Ireland; ^3^ Department of Botany Buda Campus, Hungarian University of Agriculture and Life Sciences Budapest Hungary; ^4^ Department of Evolution and Ecology University of California Davis California USA; ^5^ School of the Environment University of Queensland Brisbane Queensland Australia; ^6^ Department of Biological Sciences Louisiana State University Baton Rouge Louisiana USA; ^7^ Department of Biology University of Virginia Charlottesville Virginia USA; ^8^ School of Biosciences University of Sheffield Sheffield UK; ^9^ School of Life and Environmental Sciences University of Sydney Sydney New South Wales Australia; ^10^ Pyrenean Institute of Ecology (CSIC) Zaragoza, Jaca Spain; ^11^ School of Natural Sciences Zoology, Trinity College Dublin Dublin 2 Ireland; ^12^ Department of Biology Washington University in St. Louis St. Louis Missouri USA; ^13^ Department of Biology University of Oxford Oxford UK; ^14^ Agri‐Food and BioSciences Institute Belfast UK; ^15^ Department of Biology University of Turku Turku Finland; ^16^ Faculty of Biology and Environmental Sciences University of Helsinki Helsinki Finland; ^17^ Department of Ecology, Environment, and Plant Sciences Stockholm University Stockholm Sweden; ^18^ Departamento de Ciencias de la Vida Alcalá de Henares, Universidad de Alcalá Madrid Spain; ^19^ Institute of Ecology and Earth Sciences, University of Tartu Tartu Estonia; ^20^ Department for Physiological Diversity Helmholtz Centre for Environmental Research (UFZ) Leipzig Germany; ^21^ German Centre for Integrative Biodiversity Research (iDiv) Halle‐Jena‐Leipzig Leipzig Germany; ^22^ Department of Civil Engineering and Environmental Sciences Western Norway University of Applied Sciences Bergen Norway; ^23^ Department of Species Interaction Ecology Helmholtz Centre for Environmental Research Leipzig Germany; ^24^ Department of Natural Sciences Manchester Metropolitan University Manchester UK; ^25^ Faculty for Environmental Sciences and Natural Resource Management Norwegian University of Life Sciences Ås Norway; ^26^ Norwegian Institute for Nature Research Oslo Norway; ^27^ School of Biological, Earth and Environmental Sciences University College Cork Cork Ireland; ^28^ Department of Energy, Environment and Climate Action Arthur Rylah Institute for Environment Research Heidelberg Victoria Australia; ^29^ School of Biological Sciences Monash University Clayton Victoria Australia; ^30^ Department of Ecology, Environment and Plant Sciences Stockholm University Stockholm Sweden; ^31^ Faculty of Biology University of Marburg Marburg Germany; ^32^ Natural History Museum University of Oslo Oslo Norway; ^33^ Institute for Wildlife Management and Nature Conservation, Georgikon Campus, Hungarian University of Agriculture and Life Sciences Keszthely Hungary; ^34^ Martin Luther University Halle‐Wittenberg Halle Germany; ^35^ Department of Evolutionary Biology, Ecology and Environmental Sciences University of Barcelona Barcelona Spain; ^36^ Institute of Research in Biodiversity (IRBio), University of Barcelona Barcelona Spain; ^37^ Institute of Botany, Czech Academy of Sciences Průhonice Czech Republic; ^38^ Department of Botany, Faculty, of Science Charles University Prague Czech Republic; ^39^ Norwegian Institute for Nature Research Bergen Norway; ^40^ School of Agriculture, Food and Ecosystem Sciences University of Melbourne Melbourne Victoria Australia; ^41^ University of Talca Talca Chile; ^42^ University of Tasmania Hobart Tasmania Australia; ^43^ Ecology and Genetics University of Oulu Oulu Finland; ^44^ The National Research Centre for the Working Environment Copenhagen Denmark; ^45^ Department of Integrative Biology University of Guelph Guelph Ontario Canada; ^46^ Centre for Conservation and Ecology Genetics University of Canberra Canberra Australian Capital Territory Australia; ^47^ School of the Environment The University of Queensland Brisbane Queensland Australia; ^48^ Department of Natural Resource Sciences Thompson Rivers University Kamloops British Columbia Canada; ^49^ Manaaki Whenua—Landcare Research, a group of the Bioeconomy Science Institute Lincoln New Zealand; ^50^ Department of Plant and Microbial Biology North Carolina State University Raleigh North Carolina USA; ^51^ Department of Agricultural, Forest and Food Science University of Torino Torino Italy; ^52^ Departamento de Biología Vegetal y Ecología, Facultad de Biología Universidad de Sevilla Seville Spain; ^53^ Department of Geography King's College London London UK; ^54^ Fenner School of Environment & Society The Australian National University Canberra Australian Capital Territory Australia; ^55^ Department of Biology and IGDP in Ecology The Pennsylvania State University University Park Pennsylvania USA; ^56^ Institute of Biology/Geobotany and Botanical Garden, University of Halle Halle Germany; ^57^ Department of Evolution and Ecology University of California, Davis Davis California USA; ^58^ Lendület' Seed Ecology Research Group Institute of Ecology and Botany, HUN‐REN Centre for Ecological Research Vácrátót Hungary; ^59^ Estonian University of Life Sciences, Institute of Agricultural and Environmental Sciences, University of Tartu Tartu Estonia; ^60^ Agricultural and Biological Sciences Department Truman State University Kirksville Missouri USA; ^61^ Department of Biology Oklahoma State University Stillwater Oklahoma USA; ^62^ Biogeosciences, Faculty of Science & Technology Queensland University of Technology Brisbane Queensland Australia

**Keywords:** big data, collaborative, coordinated distributed network, data management, data product, demography, macroecology, team science

## Abstract

1. Ecological data are increasingly collected by networks of collaborators using replicated designs and methods, which can significantly improve the quality and quantity of data throughout the ecological niche and geographic range of species or communities. The coordinated generation and management of data is critical for producing datasets (Standard Data Products) across multiple sites that can be used by different researchers, over extended time periods and for multiple purposes.
2. We describe and use a Quality Assurance framework for the design, collection and production of reproducible Standard Data Products for distributed ecology projects. We identified six critical project elements of a Quality Assurance framework (QA1‐6) to produce ecological Standard Data Products with high immediate and future value.
3. We applied the Quality Assurance framework to the Plantpopnet project as a case‐study. Plantpopnet is a coordinated distributed system for population macroecology using the model species *Plantago lanceolata* . We mapped Plantpopnet activities to the Quality Assurance Framework as follows: (QA1) *Measurable objectives*: research project objectives with data requirements, (QA2) *Process control*: governance policies, (QA3) *Project specific procedures*: model organism selection and data collection protocol, (QA4) *Supporting production of high quality data*: recruitment, retention and engagement of participants, (QA5) *Data management*: data management plan and reproducible data cleaning workflow, (QA6) *Production and management of outputs*: Standard Data Products and papers.
4. Explicit use of Quality Assurance, project and data management tools together with standardised ecological methods facilitated the design, collection, maintenance and sustainability of high‐quality data products. We provide a Quality Assurance framework together with governance documents, code and data for a reproducible Standard Data Product. This framework supports a distributed funding model which can be sustainably applied to facilitate future research and applications of coordinated distributed ecology projects.

## Graphical abstract

Ecological data are increasingly collected by networks of collaborators using replicated designs and methods, which can significantly improve the quality and quantity of data throughout the ecological niche and geographic range of species or communities. We provide a Quality Assurance framework together with governance documents, code and data for a reproducible Standard Data Product for coordinated distributed ecology projects. We use the Plantpopnet team science project as a case‐study for the application of the quality assurance framework.

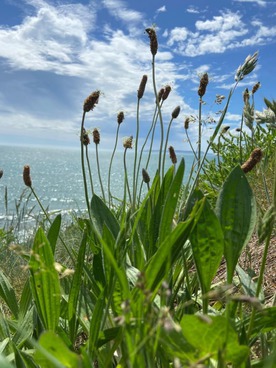

## 1 Introduction

**FIGURE 1:**
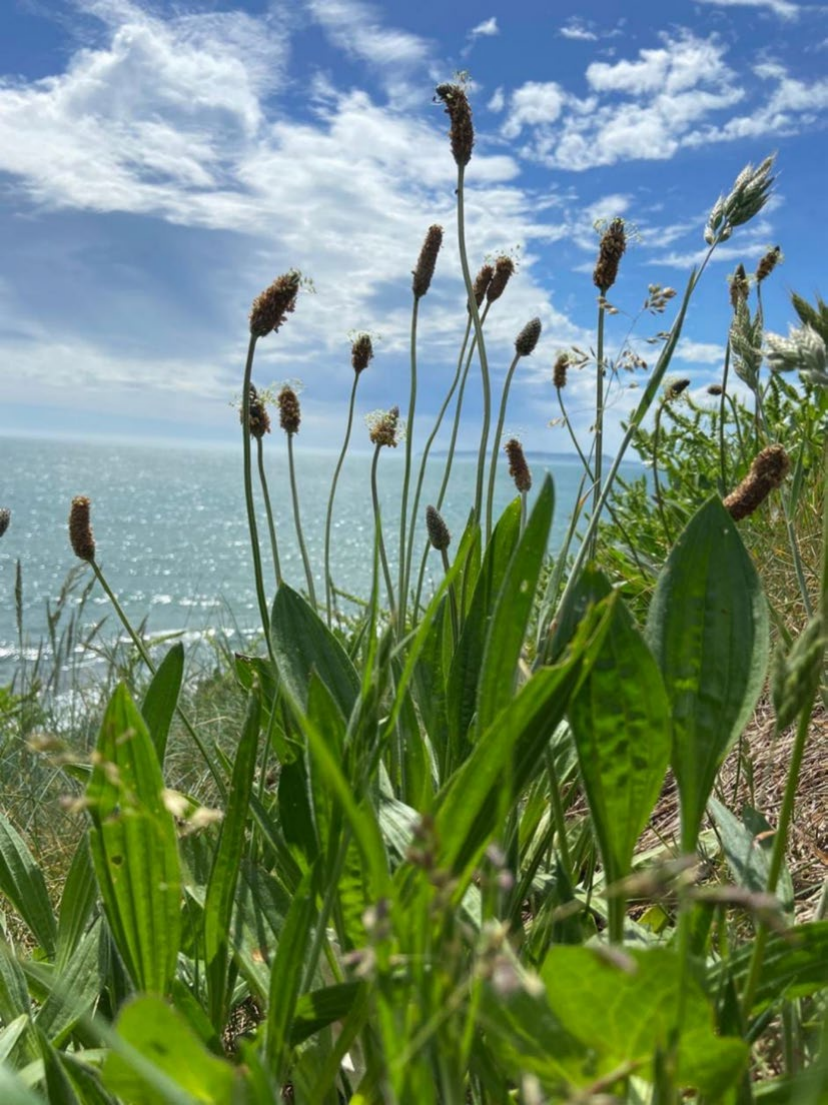
A population of *Plantago lanceolata* L. (ribwort plantain) the focal species for the Plantpopnet distributed population ecology project, at Portrane Co Dublin Ireland.

Understanding and predicting processes at large spatial extents and over time underpins ecology, evolution and conservation. Research in ecology is increasingly international, across a species' range (Buckley and Puy 2022) or ecosystem (Borer et al. 2014, Mirtl et al. 2018). Spatially extensive datasets enable the understanding of context dependency of ecological processes across sites and systems (Catford et al. 2022). However, the intensity of field work required for spatially extensive data collection has historically restricted its scope (Buckley et al. 2010; Coutts et al. 2016). One solution to this challenge is distributed ecology projects where networks of researchers address questions at large spatial extents, reducing the intensity of field work for contributors e.g., NutNet (Borer et al. 2014), GrENE‐net (Czech et al. 2022), HerbVar (Herbivory Variability Network et al. 2023), iCONNECT (Lucas et al. 2024), GLUE (Santangelo et al. 2022) and Plantpopnet, a distributed population ecology project focussed on the model species *Plantago lanceolata* L. (Figure 1).

Spatially extensive, replicable and reproducible systems can eliminate or minimise apparent context dependency arising from differences in statistical inference or methodological differences and offer an opportunity for learning about replicability and generalisability in ecology (Catford et al. 2022). We contend that Quality Assurance (QA) processes can further minimise apparent context dependency, enabling ecologically meaningful context to be more clearly discerned. QA refers to an ongoing, continuous process of monitoring, evaluating, maintaining and enhancing a product or service (QQI 2021), enabling the consistent production of a product or service that minimises defects and increases utility. QA management frameworks are commonly used across industries including environmental science (CEN 2015a; Goldberg et al. 2016; Kim et al. 2022; Sutter et al. 2015). Here we use QA principles throughout the full life‐cycle of a coordinated distributed ecology project to define objectives, ensure good governance, develop appropriate and widely applicable protocols, support participation and curate data through reproducible data cleaning, auditing, versioning and the production of Standard Data Products that can be widely used. Standard Data Products (SDP) are validated and quality assured data sets that are well documented and shareable. Research objectives such as FAIR data management (Wilkinson et al. 2016) or the creation and management of large networks such as the International Long Term Ecological Research system can be nested within a Quality Assurance system.

QA increases confidence that quality requirements will be fulfilled through a set of established procedures or processes that ensure: (QA1) *Measurable objectives* are supported by the data, where the proposed data is fit for the objectives of the project, (QA2) *Process controls* are in place. Process in this instance refers to interacting activities that use inputs to deliver an intended result. Process controls are methods and protocols implemented to ensure that the operation and control of these processes are effective, (QA3) *Project specific procedures* are used for accurate data collection, defined as specified ways to carry out activities (or processes), (QA4) *Supports for data collectors* are in place to enable collection of high quality data, (QA5) *Data management* is used to control errors and ensure data integrity during transcription, transfer and storage and (QA6) *Production and dissemination of high quality outputs*, with outputs defined as the result of a process (CEN 2015a). Quality Control (QC) includes technical checks to ensure the product (in this case, data) fulfils quality requirements. Previous work on quality aspects of ecological data collection have tended to focus on individual components of a QA system, such as data management (e.g., Borer et al. 2009; Kim et al. 2022) or the development of modelling standards (e.g., Araújo et al. (2019)), protocols, or networks; but see Borer et al. (2014) for an integrated approach.

Distributed projects often involve a common protocol for data collection, standard methods and consistent reporting of data using templates. These are useful elements of a more comprehensive QA process, but are not sufficient. Distributed collaborative research (team science) in ecology typically uses a common experimental or observational protocol deployed across multiple locations by many researchers (Alexander et al. 2011; Borer et al. 2014; Henry and Molau 1997) run in parallel across geographic scales to test ecological questions (Pastor et al. 2020). Data, samples or both, are collected by participants from multiple sites and used to answer research questions at both the fine grain needed to understand local ecological processes and the large spatial extent needed for contextualisation and generalisation (Wiens 1989). Team science can widen participation in ecological research, enable sharing of knowledge, expertise and costs among contributors, reduce greenhouse gas emissions and other costs associated with international research (Diagne et al. 2021), enable simultaneous sampling across large areas, and increase the resilience of research to global shocks. However, there are significant challenges in ensuring that the resulting data have sufficient quality, longevity, reproducibility, accessibility and utility to answer the targeted research questions, as well as future value.

The compilation of data collected by collaborating researchers at large spatial and temporal scales has a long history (Heberling et al. 2021; Henry and Molau 1997). However, managing effective distributed networks goes beyond implementing data quality checking. Networks must ensure quality is achieved across project management, team engagement, data curation and the reproducibility of data products used as inputs to research projects (Borer et al. 2014; Maund et al. 2022). Replicability in coordinated distributed networks is built into the observational or experimental protocols, standardised across sites. However, methods for curating and generating reproducible data products from multiple contributions are rarely presented (Hampton et al. (2013); Buckley et al. (2025); but see Herbivory Variability Network (2023) and Lind (2016)).

Here we describe a general QA framework which provides a template for the design of distributed ecology projects. We illustrate this QA framework for a distributed population ecology network, Plantpopnet [www.plantpopnet.com], and demonstrate its utility from inception to the production of a Standard Data Product (SDP) ready for use by data consumers (researchers in this case). The production of SDPs facilitates reproducibility of analyses and enables citation of specific instances of evolving data sources. To illustrate our QA approach, we mapped Plantpopnet activities to the QA framework. We provide a QA workflow for reproducible SDPs and assess error rates across sites.

### 1.1 Plantpopnet Case‐Study

**FIGURE 2:**
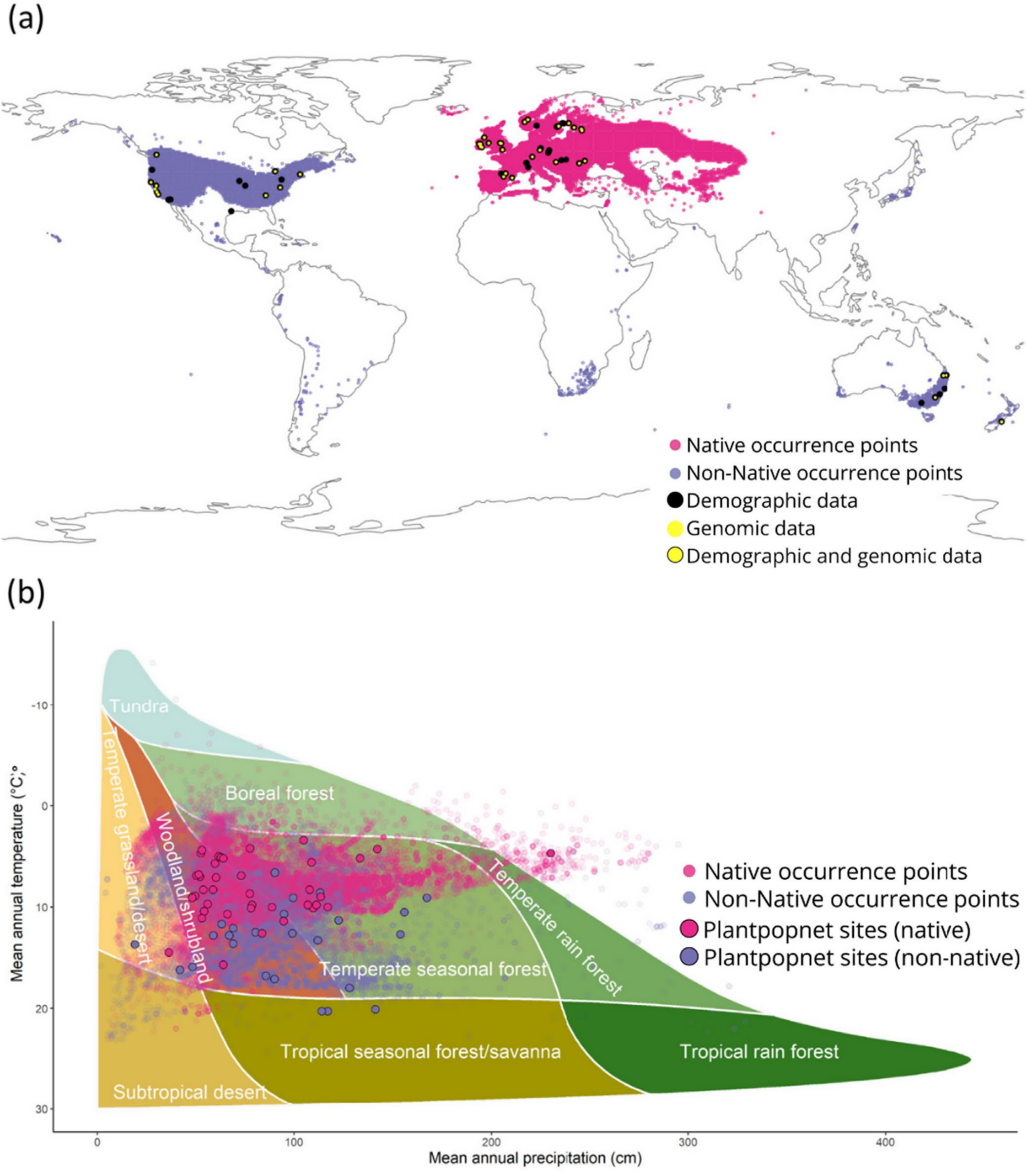
(a) Map of 64 demographic data collection sites with at least one year of demographic data and 37 sites for both genomic and demographic data (Smith et al. 2020) throughout the native (small pink points) and non‐native (small purple points) range of *Plantago lanceolata* (as recorded on GBIF (GBIF 2022) & BIEN (Enquist et al. 2016)). (b) an indicative position of the data collection sites in Whittaker Biomes. Each pink (native) and purple (non‐native) point represents a data collection site and its corresponding biome. One population in Norway lies outside the definition of Whittaker's biome due to high local precipitation relative to its mean annual temperature. Climatic data in (b), shown as transparent points for *P. lanceolata* observations in GBIF and BIEN, is from WorldClim 2 (Fick and Hijmans 2017) and is produced using ‘plotbiomes’ (Stefan and Levin 2019).

Plantpopnet uses the perennial short‐lived herb *Plantago lanceolata* L. (Plantaginaceae) as a model organism to understand range‐wide biotic and abiotic drivers of population change (Penczykowski and Sieg 2021). Since 2014, 75 site coordinators in 17 countries and 3 continents have submitted data from 65 populations (Figure 2). The Plantpopnet project enables the development and testing of methods for a biogeographical approach to population ecology based on a unique, spatiotemporally replicated demographic dataset of plants and animals that can be applied to other systems (Alexander et al. 2012; Baudraz et al. 2025; Buckley and Puy 2022; Kuiper and Bos 1992; Penczykowski and Sieg 2021; Sagar and Harper 1961; Smith et al. 2020; Villellas et al. 2021).

### 1.2 Quality Assurance Framework

A QA framework for coordinated, distributed ecology projects was adapted from fundamental principles and requirements of ISO Quality Management Systems (CEN 2015a, 2015b). We outline the application of each QA objective to Plantpopnet and explain how the framework evolved in line with QA guidelines for adaptability and flexibility (an overview is provided in Table 1).

**TABLE 1:** Framework for Quality Assurance of distributed ecology projects. We outline general Quality Assurance objectives (QA1–QA6), measurable indicators for Quality Control, specific examples of implementation of objectives for Plantpopnet (PPN1–PPN10, PPN referring to a Plantpopnet activity) and Quality Control (QC1–QC9) evidence is detailed for Plantpopnet activities. More information is available in the Supporting Information.

Quality Assurance objectives	Measurable indicators for Quality Control	Plantpopnet activities (timeline of implementation)	Quality Control evidence
QA1: *Measurable objectives* supported by data	Number of research questions/research objectives % of research questions/objectives supported by data collected	PPN1: Development of research project abstracts with data requirements (2014)	QC1. Development & refinement of questions at workshops (2014, 2017, 2021)
QA2: *Process controls*	Number of policies supporting governance, recruitment and retention of participants, funding. % of active projects following network governance policy Number of steering committee meetings	PPN2: Policies for Network Governance (Supporting Information S1: 1.1), Authorship (Supporting Information S1: 1.1) (2014)	QC2. Review & updates at steering committee meetings
		PPN3: Policy for funding (Supporting Information S1: 1.3) (2019)	
QA3: *Project‐specific procedures*	Observational/experimental system in place (pass/fail) Protocol in place (pass/fail) % of sites applying protocol correctly Number of protocol reviews	PPN4: Model organism selection (2014)	QC3. Selection framework developed (2014)
		PPN5: Data collection protocol (2014)	QC4. Network review of protocol (2014, 2019)
QA4: *Supports for data collectors* to produce quality data	Number of participants and sites recruited % participant and site retention Number of communications with participants Number of training events and resources provided % of participants engaged in training	PPN6: Recruitment, retention and engagement of participants for long term research (2014, 2015, 2016)	QC5. Analysis of participant & site data, including exit data. Review at participants' workshop (2021).
QA5: *Data management* to control errors and ensure data integrity during transcription, transfer and storage	Data Management Plan in place (pass/fail)	PPN7: General data management and storage processes (2017)	QC6. Steering Committee reviews of data management plan
		PPN8: Reproducible data cleaning workflow & SDPs	QC7. Internal peer review of workflow & scripts (2019, 2020)
QA6: *Production & dissemination of high quality outputs*	Number of Standard Data Products (SDPs) Number of citations to SDPs Number of publications or other outputs using SDPs Number of citations to outputs using SDPs Number of SDP downloads	PPN9: Publication, dissemination and versioning of Standard Data Products (SDPs)	QC8. Data audit of SDPs
		PPN10: Publication of papers & reports (Baudraz et al. 2025; Buckley et al. 2019; Morales et al. 2021; Smith et al. 2020; Villellas et al. 2021)	QC9. Internal & external manuscript peer review

#### 1.2.1 QA1: Measurable Objectives Supported by Data

Clear definition of research questions and appropriate research design is imperative (Hayward et al. 2015). Co‐design and collaborative processes are particularly useful in distributed ecological networks for defining research objectives (Table 1, PPN1). Engagement of participants ensures that objectives and procedures are robust and that the commitment of participants is secured (McCord et al. 2022). A core set of common research objectives can be supplemented with ‘add‐on’ research objectives that may require additional data collection and/or a more limited number of sites for specific observations or experiments (Borer and Seabloom 2025). For example, Villellas et al. (2021) used core data collected through the standard Plantpopnet protocol (46 populations) with add‐on protocols at a subset of sites for seed collection (15 populations) subsequently used for an experiment. Add‐on studies have many benefits, including allowing participants to enhance their own research by defining research questions, building on existing data and reducing costs. Add‐on studies increase data collection, test new research questions, improve efficiency through utilising existing protocols and methodologies, and enhance collaboration and knowledge exchange.

Importantly, research objectives must be explicitly linked to sampling design, spatial coverage, temporal duration and planned analytical approaches. The ability of a network to address broad‐scale ecological questions depends on whether sampled sites adequately represent the environmental, geographic and demographic gradients relevant to the study objectives. Under‐representation of particular regions, habitat types or environmental conditions can limit the generalisability of findings and constrain inference regarding species responses across their full range (Borer and Seabloom 2025). Similarly, temporal coverage must be sufficient to capture the ecological processes of interest (Fraser et al. 2013) and support the statistical or modelling approaches proposed. During network design, objectives should therefore be evaluated against expected sampling coverage and data structure to ensure that the resulting dataset is capable of supporting robust inference. Where gaps in coverage remain unavoidable, these limitations should be explicitly recognised, and research questions, analytical methods or recruitment efforts adapted accordingly.

#### 1.2.2 QA2: Process Controls

Clear governance is needed to ensure transparent management of a coordinated distributed collaborative network (e.g., top‐down versus bottom‐up management (Aubin et al. 2020)) and should be developed early in the project and revised regularly. Decision making, funding, authorship and data availability processes ensure that the responsibilities and benefits of participation are clear to participants. Decision making may be undertaken by a subset of participants. However, careful consideration should be paid to geographic distribution across regions of research interest, a diverse suite of professional networks, gender balance, under‐represented groups and countries, early career participants and multidisciplinary expertise (Supporting Information S1: 1.1).

Process controls include management of funding. A variety of funding mechanisms can be used such as grants for: collaborative workshops, research coordination, data collection at individual sites and individual research questions. This distributed funding model enables flexible research and encourages wider participation by researchers. For Plantpopnet, a distributed funding model was adopted with individual sites largely self‐funded. We conservatively estimated a total of 1.2 person years per year of operation has been invested in the collection and curation of core demographic data (equivalent to 8.35 years of one full‐time person over 2014–2021, Supporting Information S2).

#### 1.2.3 QA3: Project‐Specific Procedures

Outlining and confirming project procedures enhances data quality (Brown and Williams 2019). Strong project design with clear, shared project objectives and methodology enables the production of long‐term, reproducible data which can often address multiple novel research questions (Borer et al. 2014; Fraser et al. 2013). Choice of the system of study is fundamental to the achievement of objectives, and the potential to leverage core data for other kinds of questions both in situ (local sites) and *ex situ* (growth chambers, common gardens) should be considered.

##### 1.2.3.1 PPN4: Model Organism Selection

Candidate species were assessed against indicators of key ecological and evolutionary attributes using expert knowledge and published literature before *P. lanceolata* was selected (Penczykowski and Sieg 2021) (Table 2).

**TABLE 2:** Key considerations for protocol development.

Considerations	Justification for Plantpopnet
Provision of a clear methodology for individual plant level demographic monitoring over time	Persistence in plots for 3–5 years across a broad environmental gradient Adaptable to different population densities and site conditions Ease of finding transects, plots and individual plants from year to year Minimally invasive monitoring to reduce the impact on population processes Ensure minimum amounts of permits required, specifically consideration of permits required to work in sensitive areas
Accessibility of methods to maximise participation of voluntary participants	Flexible choice of site location Inexpensive methodology Clear and easy to follow methodology with data collection template spreadsheets Up to 3 person/days time commitment per year for annual census Modular design of core data collection protocol with optional ‘add‐on’ opportunities for additional data

##### 1.2.3.2 PPN5: Data Collection Protocol

The first version of the protocol was tested in 2014, revised, and provided to participants in 2015 (V1.01). Second and third revisions of the protocol were provided on FigShare for transparent version control, long‐term archiving and citation purposes (Buckley et al. 2019).

Key considerations for protocol development included a clear methodology for demographic monitoring over time and accessibility of methods to maximise sustained participation (Table 2). Trade‐offs arose as data for answering some demographic questions was deemed too challenging and/or time‐consuming to collect. By prioritising data requirements, we developed a core protocol that every site must complete with additional data collection encouraged through optional ‘add‐on’ studies. For example, the annual demographic census for Plantpopnet is obligatory, but collection of leaves for genetic analysis or seed for experiments was chosen by participants as ‘add‐on’ elements depending on their research interests and capacity. This approach ensures a comprehensive core dataset combined with the agility to ask new research questions according to participant interests.

#### 1.2.4 QA4: Supporting Data Collectors to Produce Quality Data

The success of a spatially distributed, long‐term team science project depends strongly on the network of participants; however, the methodology for achieving group engagement and retention is rarely described. Having clear roles and structures, workshops, training and support for members, goals that all members can contribute to, and having members at different career stages has been identified as contributing to successful team science projects (Cheruvelil et al. 2014; Love et al. 2021). Large‐scale spatial projects necessarily require a diverse range of scientists from various geographic locations.

##### 1.2.4.1 PPN6: Recruitment, Retention and Engagement of Participants for Long Term Research

Several dimensions of sustainability were considered in the design of the research network including: long‐term data collection, environmental sustainability, resources and long‐term capacity to maintain data collection. Some objectives were achievable within a single or few years of data collection whereas other objectives required long‐term (decadal) timescales. The need for sustainability influenced the recruitment strategy. To encourage diverse participation the steering group consisted of researchers of different career stages, genders and geographic locations who approached potential participants, and promoted the network on social media and at conferences. We recruited participants of different career stages to ensure both short‐ and longer‐term objectives were achievable. We encouraged participants to choose sites close to their place of work or home for ease of sustained data collection. Most participants were recruited in 2015 and 2016 with small numbers of sites joining after this time (Supporting Information S3). The measures/strategies that were taken to maximise participation resulted in 34% of 65 original sites still actively collecting data as of 2024.

Reasons for site abandonment (*n* = 16) included: change in site coordinator circumstance (38%), mass mortality of censused plants (19%), landowner's request (13%) and destruction of the site (accidental or otherwise) (25%). The highest rate of abandonment of sites occurred after 2 years of data collection, where 14% of sites with 2 years of data were abandoned (Supporting Information S4).

**FIGURE 3:**
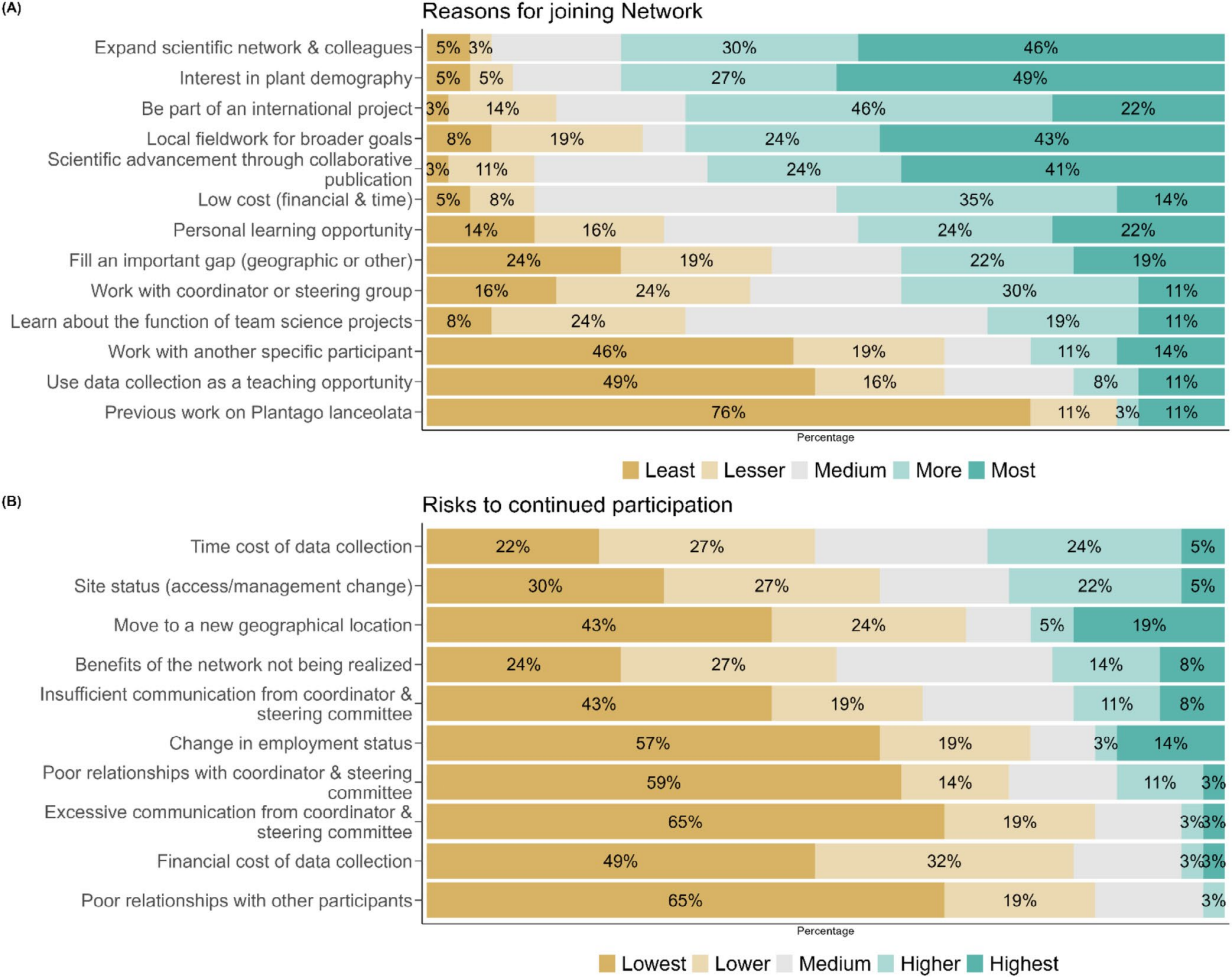
(A) Reasons for joining Plantpopnet ranked from least important to most important & (B) Risks to continued participation in the Plantpopnet network ranked from lowest risk to highest risk. Responses are ordered by most important (using ‘more’ and ‘most’ categories) and highest risk (using ‘higher’ and ‘highest’ categories) respectively.

In 2018 site participants (*n* = 41) were surveyed online to understand the composition of the network and participants' motivations for joining a team science project (as in García et al. (2019)). 90% of participants completed the survey (see Supporting Information S5 for full 10 question list). Highly ranked reasons for joining included interest in demography (49% ranked it as most important), collaborative research and scientific advancement (41%), personal learning (22%) and network interactions (46%) achievable for relatively low commitment of time, cost and travel (14%) (Figure 3A).

Participants perceived the greatest risks to continuation to be changes in their employment (14%) and geographic location (19%), which is in accordance with the primary observed reason for site abandonment (change in participant circumstances) (Figure 3B; Supporting Information S5).

#### 1.2.5 QA5: Data Management to Control Errors and Ensure Data Integrity

Units of measurement, file naming and location of stored data should be consistent and agreed prior to commencing (Michener 2018). Detailed metadata with a README file, clearly and consistently labelled files in appropriate folders, clear steps for data processing and utilising centralised, community‐maintained controlled‐access repositories for metadata and code all contribute to effective data management (Jenkins et al. 2023). To control errors in the data cleaning and analysis processes, code should be written in a manner that enables clear communication, and a formatting style that is easily understandable and consistent (Buckley et al. 2025; Filazzola and Lortie 2022). Specialised apps can be used to control data collection errors, e.g., HerbVar has an app to train herbivory estimation (www.zaxherbivorytrainer.com) and PhenObs (phenological network using botanical gardens, https://www.idiv.de/en/phenobs) uses an app to standardise data collection, ensuring consistency in data recording.

##### 1.2.5.1 PPN7: General Data Management and Storage Processes

The primary investigator at each site (i.e., site coordinator) is responsible for adhering to the protocol (Buckley et al. 2019) and providing digital data. Data files can be accompanied by images, physical samples, maps and notes.

**FIGURE 4:**
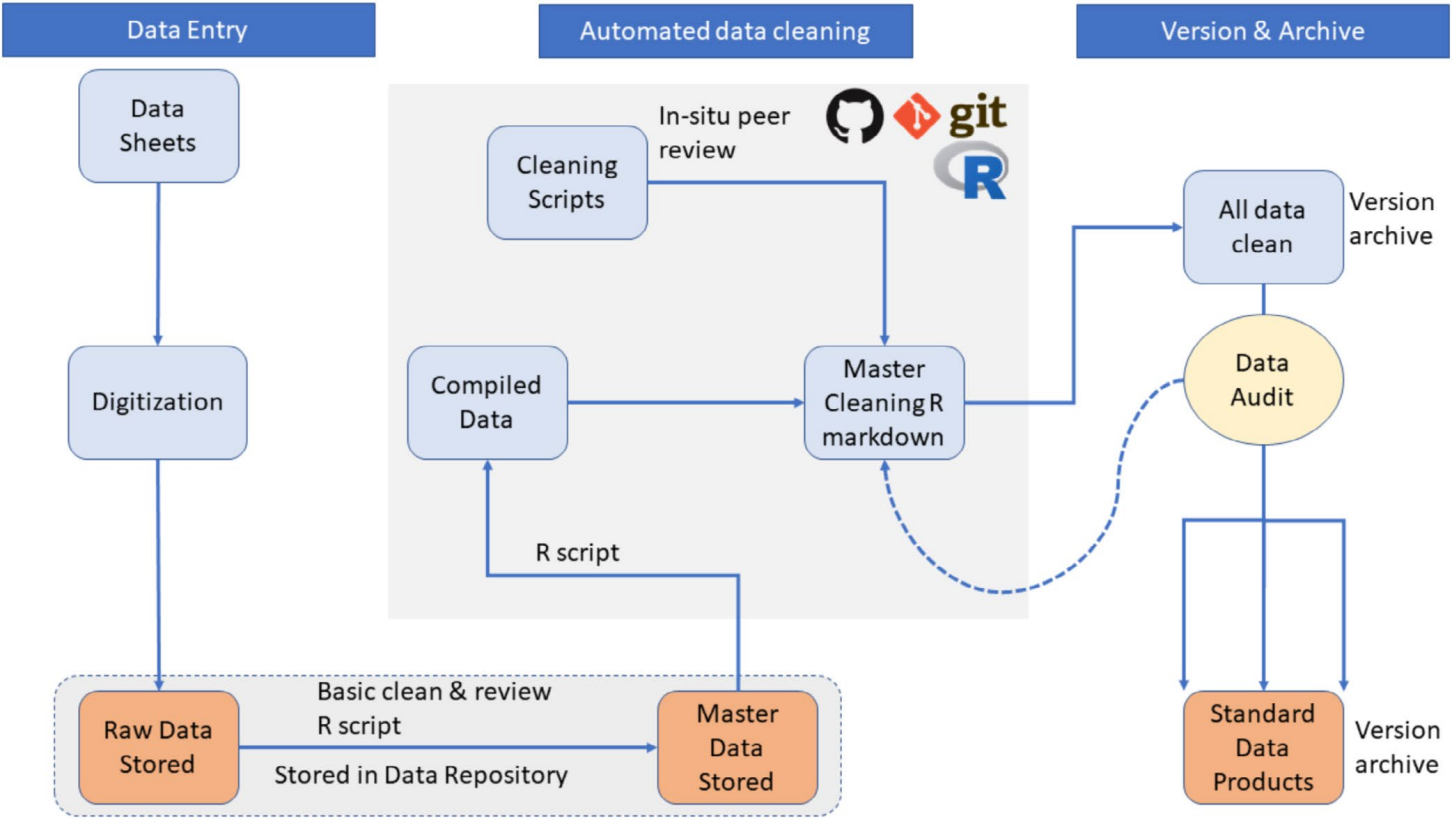
Data management workflow from point of creation by site coordinators to production of Standard Data Products (SDPs). Raw data is converted to master data, which is cleaned via a semi‐automated system of R scripts and maintained and stored using Git and the online repository Github (icons under Automated data cleaning represent software tools). ‘Cleaned’ data is subjected to an audit before final export to SDPs. A data audit determines if errors were introduced through the cleaning scripts.

In 2018, a data management plan for Plantpopnet was developed (Supporting Information S1: 1.4) based on the data management guidelines developed by Borer et al. (2009), detailing file storage, naming, data sharing and access procedures and a checklist for the data management workflow (Figure 4). A key challenge is recording changes made to the raw data before analysis. We took a scripting approach to recording data changes, to ensure transparency, to enable changes to be reversed, and to enable a record of edits to be generated. This approach ensured that the raw data could be converted to an SDP in a reproducible way, following recent data curation reproducibility guidelines (Buckley et al. 2025). Raw data files were not altered, thus any errors arising in compiled datasets could be traced back and queried against the original submission.

Initially, raw data files were manually verified to ensure correct protocols were followed, that data were consistent and complete, and the established criteria for QC results were met. Each verified raw file was then stored as a ‘master’ copy. Standardised master data for all sites and all years were combined, and the resulting dataset was error checked using scripts (Figure 4). Finally, the standardised dataset was used to produce SDPs corresponding to cumulative census years and distributed for analysis.

R scripts, R Markdown files detailing processes and changes, and data products associated with the cleaning process were maintained using version control software Git and online repository Github. GitHub stores the online working repository and version history of the cleaning scripts, whereas the scripts available on Zenodo under the Data Availability Statement are archived for release and reproducibility with this manuscript.

##### 1.2.5.2 PPN8: Reproducible Data Cleaning Workflow and Standard Data Products (SDPs)

As datasets increase in size, so does the complexity of the curation process. A working group was formed to assemble scripts to identify and solve errors in the data. Each script tackled specific variables and was incorporated into an annotated markdown file. Project management was through GitHub, which was used to track errors and solutions and included internal peer review of scripts.

When passing large datasets through a series of scripts, it is possible to introduce new errors; therefore, a check of the standardised data is a vital step. A random subset of sites per year from the draft SDP was cross checked against the uncleaned master data using the R package ‘compareDF’ (Joseph 2021), enabling identification of differences between two similarly structured datasets. Sites were randomly selected using R's random number generator, with a fixed starting value (or ‘seed’) to ensure reproducibility of the sampling process.

All ten sites audited from the Y0 (Year 0, first year of data collection) SDP had some level of modification to the raw data: affecting 2 to 199 rows per site, representing 1.2% to 98% of rows in a site with at least one cell edited. Most were minor formatting or continuity errors, e.g., cells with ‘NA’ were converted to blank cells. However, six sites had substantial changes, with three of these sites requiring removal of some data. Substantial changes were defined as changes to data values, including removal of data not included in the protocol, correcting units and protocol deviations, such as recording multiple values in a single cell. The most common errors included columns which were removed or re‐labelled from the protocol template data file, values outside defined ranges or incomplete records. Corrections in these six sites affected 2–68 rows (representing 1.2%–32% of rows in a site with at least one cell edited). Corrections were recorded as annotations in the data checking script. There were no instances of errors introduced through the data cleaning process itself. The data check also incorporated a review of each site and their data submissions, highlighting sites missing from the Y0 (v1.0) dataset.

#### 1.2.6 QA6: Production & Dissemination of High Quality Outputs

Sharing data outputs that support publications allows for replication, and sharing data within a network and beyond allows for increased standardisation as the data is re‐used (Parr and Cummings 2005). High‐quality outputs are produced through rigorous peer review processes and data audits to ensure QC (Ferreira et al. 2016).

##### 1.2.6.1 PPN9: Publication, Dissemination and Versioning of SDPs

Each SDP combines data from all sites. As new data become available, or new cleaning procedures are implemented, new versions of each SDP are produced, named and old versions archived. For example, the Y0 SDP contains data for all sites from the first year of data collection, the Y1 SDP contains data for 2 years, enabling one demographic transition to be analysed. A record of changes from version to version is maintained for comparison. The first Plantpopnet SDP (Y0v1.3) is provided with this paper, including a summary of the data at site level.

##### 1.2.6.2 PPN10: Publication of Papers, Reports

To avoid publication conflicts and enhance collaboration, all uses of the (unpublished) core demographic data across multiple sites require approval from the steering group. A participant submits an abstract for approval (using the abstract template provided, Supporting Information S1: 1.5) outlining the research question, intended use of data products and any additional data to be collected. Potential authorship is identified and normally includes all data contributors as well as other collaborators with key expertise. An authorship guidelines document is used to determine authorship processes (Supporting Information S1: 1.2). To ensure quality, all manuscripts produced using Plantpopnet data are internally reviewed by participants who have contributed data. A record is kept of manuscripts and their stage towards completion and updated every 12 months.

## 2 Discussion

We developed and applied a QA management framework to ensure that the data from coordinated, distributed ecology projects have sufficient quality, longevity, reproducibility, accessibility and utility for current and future use. Applying the QA framework to the Plantpopnet project, we show how coordinated actions can be structured, reviewed and checked. For sustained benefits to be realised, we emphasise the importance of data management processes, including the reproducibility of data‐cleaning and the production of SDPs. While other projects have used individual QA or QC components, we have implemented a full project lifecycle approach to QA that supports better reproducibility of the many different components of a successful coordinated distributed network project from inception to data production and sustainable use.

Reproducible methods for SDP production are important given the heterogeneity in the quality of the data submitted, the use of the resulting data by many different researchers, and the expected longevity of the unique datasets produced. QA and QC principles applied to data curation supported consistent production of SDPs to be used for further analysis. The SDPs will be critical inputs into ongoing data analysis projects within the network and as SDPs are progressively publicly released, they will become an important resource for the plant demography and ecological research community. By releasing the Y0 SDP together with the raw data and scripts for data cleaning we hope to inspire wider uptake of this approach to reproducible data cleaning in ecology (Buckley et al. 2025). QA is consistent with the emergence of the Open Science Framework (Foster and Deardorff 2017; O'Dea et al. 2021) and the FAIR (Findability, Accessibility, Interoperability and Reusability) Data Management principles (Wilkinson et al. 2016), which provide a platform and tools needed to radically improve transparency, reproducibility and reliability in science. In adhering to open data principles, it is also important to consider local and jurisdictional‐level laws and customs, to ensure local and Indigenous communities maintain data rights (Carroll et al. 2020).

Team science in ecology requires a range of new methods and ways of working to maximise benefits from collaborative, distributed data collection and analysis. Contributors have the opportunity to provide data, a different perspective and further spatial extent (Borer et al. 2023; Czech et al. 2022; Herbivory Variability Network et al. 2023; Lucas et al. 2024; Santangelo et al. 2022). Collecting consistent field data across multiple network participants is challenging however, and although a detailed protocol and data collection template were provided, checks of the cleaned data identified that logistical issues and differences between ecosystems in the field contributed to deviations from the template. We found that the most frequent discrepancies in the raw data were basic structural and formatting deviations, commonly from modifications to the template for field use. In isolation, these inconsistencies seem trivial, but in a large dataset they can be difficult to manage. The need for post‐collection processing can be minimised through careful design of field data‐sheets to maximise their utility and minimise the potential for incorrect data entry (Supporting Information S6). Utilising an app for data collection could also help to prevent incorrect data entry, such as transcription errors, e.g., the PhenObs network (Sporbert et al. 2022).

A wide geographic distribution of sites was achieved for Plantpopnet, but important spatial gaps became apparent. The eastern and southern parts of the native range and arid areas are under‐sampled (Figure 2). Language capacity, lack of scientific institutions and networks, research culture differences, incomplete occurrence data and resource limitations may have hindered recruitment of participants. Language barriers can seriously limit knowledge sharing through barriers of evidence generation by non‐English speakers and the global synthesis of evidence in different languages (Amano and Berdejo‐Espinola 2025). Future efforts to identify potential collaborators and funding strategies to support participants in targeted locations may be needed to fulfil the aims of better representing the full extent of biotic and abiotic niche dimensions of this species. Wider availability of automated translation tools will enable future networks to more effectively reach potential collaborators throughout the species' range. Institutional partnerships and the co‐design, emergence and recognition of benefits of being part of coordinated distributed networks for individual researchers and their institutions should enable future networks to more effectively recruit and retain members in key locations. Currently benefits include co‐authored papers, access to important data‐sets, access to collaborative networks and international recognition; however, an understanding of different research and institutional cultures may enable additional benefits to be identified or co‐created.

### 2.1 Funding Network Science

Plantpopnet operates with a distributed funding model where individual sites are funded by network participants. Central funding has primarily been used to operate the digital infrastructure needed for the network. However, once the outputs are published, the data repository and curation systems become public goods, opening the possibilities of freeloading, where anyone can benefit (Neylon 2017). Centralised funding is common across shared infrastructures; however, sustainability models need to adapt and change over the life cycle of an infrastructure, reflecting the changes in the community that uses the service (Neylon 2017). Making the data curation and cleaning tools available to researchers within and external to the network reduces the need for centralised data curation funding, and it may be beneficial to move to an alternative funding model to ensure the longevity of the network. For example, a shift to sources that fund specific uses of the data rather than funding general data collection and curation. Securely archiving open access data together with strong documentation on protocols under which data were collected and curated can mitigate the risk of data loss on conclusion of funding streams.

### 2.2 Serendipitous Benefits

Plantpopnet has displayed an unanticipated robustness to global shocks. In 2020, it was expected that elective projects like Plantpopnet would suffer from the pressures and workloads brought about during the Covid‐19 pandemic. Just 6 of 43 active sites were unable to sample in 2020 due to the pandemic. Surprisingly, 6 new sites joined the network in 2020, more than in any of the previous 3 years (Supporting Information S3). The key factors enabling this resilience to shock were the proximity of sites to the researchers' base and the ease of sampling.

Environmental sustainability has been an achievement of the network. Carbon costs of undertaking research are under increasing scrutiny (e.g., ALLEA (2022); Baer (2018, 2019); The Wellcome Trust (2021); Wynes et al. (2019)) and in future, environmental sustainability may need to be addressed for research ethics approval and funding.

## 3 Conclusions

An explicit QA framework for management of multi‐site, multi‐participant collaborative data collection networks can support sustained engagement of participants and robustness of data collection and dissemination. The most important risks to continued data collection were instability in the location and employment of participants. This needs to be considered for the design of future networks where long term datasets are to be collected. One of the strengths of Plantpopnet was the inclusion of participants as independent site coordinators from a wide range of career stages. While changes in personal circumstances were more likely in early career participants, some maintained involvement through collection of different types of data (e.g., genetic samples), setting up of new sites, or through recruitment of collaborators to maintain their original site.

The project management and data curation skills needed to progress spatially distributed ecology projects are not commonly outlined or transferred as part of undergraduate and post‐graduate courses or continuing professional development. These skills are highly transferable and in demand within academia and beyond. By making project management transparent and central to the design and implementation of a collaborative, distributed team science project, we hope to inform the structure and design of future projects as well as to stimulate debate and the development of new methods.

## Author Contributions

**Yvonne M. Buckley:** conceptualization (equal), data curation (lead), formal analysis (lead), funding acquisition (lead), investigation (equal), methodology (equal), project administration (lead), resources (lead), software (equal), supervision (lead), validation (equal), visualization (supporting), writing – original draft (lead), writing – review and editing (equal). **Alain Finn:** data curation (equal), formal analysis (equal), methodology (equal), project administration (equal), writing – original draft (equal), writing – review and editing (equal). **Aoife Molloy:** data curation (equal), formal analysis (equal), investigation (equal), project administration (equal), writing – review and editing (equal). **Anna Mária Csergő:** conceptualization (equal), data curation (equal), investigation (equal), methodology (equal), project administration (equal), resources (equal), writing – review and editing (equal). **Elizabeth Crone:** conceptualization (equal), investigation (equal), methodology (equal), project administration (equal), writing – review and editing (equal). **Annabel L. Smith:** data curation (equal), investigation (equal), methodology (equal), resources (equal), software (equal), writing – review and editing (equal). **Bret D. Elderd:** conceptualization (equal), data curation (equal), investigation (equal), methodology (equal), project administration (equal), resources (equal), writing – review and editing (equal). **Caroline McKeon:** data curation (equal), investigation (equal), resources (equal), software (equal), writing – review and editing (equal). **Deborah Roach:** conceptualization (equal), data curation (equal), investigation (equal), methodology (equal), project administration (equal), resources (equal), writing – original draft (equal), writing – review and editing (equal). **Dylan Z. Childs:** conceptualization (equal), data curation (equal), formal analysis (equal), investigation (equal), methodology (equal), project administration (equal), resources (equal), writing – review and editing (equal). **Glenda M. Wardle:** conceptualization (equal), data curation (equal), investigation (equal), methodology (equal), project administration (equal), resources (equal), writing – original draft (equal), writing – review and editing (equal). **María B. García:** conceptualization (equal), data curation (equal), investigation (equal), methodology (equal), project administration (equal), resources (equal), writing – review and editing (equal). **Maude Baudraz:** data curation (equal), investigation (equal), methodology (equal), project administration (equal), resources (equal), software (equal), writing – review and editing (equal). **Rachel M. Penczykowski:** conceptualization (equal), data curation (equal), investigation (equal), methodology (equal), project administration (equal), resources (equal), writing – review and editing (equal). **Roberto Salguero‐Gómez:** conceptualization (equal), data curation (equal), investigation (equal), methodology (equal), project administration (equal), resources (equal), writing – review and editing (equal). **Ruth Kelly:** data curation (equal), investigation (equal), writing – review and editing (equal). **Satu Ramula:** conceptualization (equal), data curation (equal), investigation (equal), methodology (equal), project administration (equal), resources (equal), writing – review and editing (equal). **Anna Liisa Laine:** conceptualization (equal), data curation (equal), investigation (equal), methodology (equal), project administration (equal), resources (equal), writing – review and editing (equal). **Simone Blomberg:** conceptualization (equal), methodology (equal), project administration (equal), writing – review and editing (equal). **Johan Ehrlen:** conceptualization (equal), data curation (equal), investigation (equal), methodology (equal), project administration (equal), resources (equal), writing – review and editing (equal). **Jesús Villellas:** conceptualization (equal), data curation (equal), investigation (equal), methodology (equal), project administration (equal), resources (equal), writing – review and editing (equal). **Aveliina Helm:** data curation (equal), investigation (equal), writing – review and editing (equal). **Christiane Roscher:** data curation (equal), investigation (equal), writing – review and editing (equal). **Krista Raveala:** data curation (equal), investigation (equal), writing – review and editing (equal). **Liv Norunn Hamre:** data curation (equal), investigation (equal), writing – review and editing (equal). **Lotte Korell:** data curation (equal), investigation (equal), writing – review and editing (equal). **Francis Q. Brearley:** data curation (equal), investigation (equal), writing – review and editing (equal). **Siri Lie Olsen:** data curation (equal), investigation (equal), writing – review and editing (equal). **Astrid Wingler:** data curation (equal), investigation (equal), writing – review and editing (equal). **Joslin L. Moore:** data curation (equal), investigation (equal), writing – review and editing (equal). **Ayco J. M. Tack:** data curation (equal), investigation (equal), writing – review and editing (equal). **Anna Bucharova:** data curation (equal), investigation (equal), writing – review and editing (equal). **Olav Skarpaas:** data curation (equal), investigation (equal), writing – review and editing (equal). **Judit Bodis:** data curation (equal), investigation (equal), writing – review and editing (equal). **Aldo Compagnoni:** data curation (equal), investigation (equal), writing – review and editing (equal). **Sergi Munne‐Bosch:** data curation (equal), investigation (equal), methodology (equal), writing – review and editing (equal). **Zuzana Munzbergova:** data curation (equal), investigation (equal), writing – review and editing (equal). **Joachim Töpper:** data curation (equal), investigation (equal), writing – review and editing (equal). **Peter Vesk:** data curation (equal), investigation (equal), writing – review and editing (equal). **Melanie Morales:** data curation (equal), investigation (equal), writing – review and editing (equal). **Lizzie Wandrag:** data curation (equal), investigation (equal), writing – review and editing (equal). **Marjo Saastamoinen:** data curation (equal), investigation (equal), writing – review and editing (equal). **Martin Andrzejak:** data curation (equal), investigation (equal), writing – review and editing (equal). **Pil Rasmussen:** data curation (equal), investigation (equal), writing – review and editing (equal). **Christina Caruso:** data curation (equal), investigation (equal), writing – review and editing (equal). **Richard Duncan:** data curation (equal), investigation (equal), writing – review and editing (equal). **John Dwyer:** data curation (equal), investigation (equal), writing – review and editing (equal). **Matthew Coghill:** data curation (equal), investigation (equal), writing – review and editing (equal). **Ronny Groenteman:** data curation (equal), investigation (equal), writing – review and editing (equal). **Meelis Pärtel:** data curation (equal), investigation (equal), writing – review and editing (equal). **William K. Petry:** data curation (equal), investigation (equal), writing – review and editing (equal). **Michele Lonati:** data curation (equal), investigation (equal), writing – review and editing (equal). **Javier Puy:** data curation (equal), investigation (equal), writing – review and editing (equal). **Jane Catford:** data curation (equal), investigation (equal), writing – review and editing (equal). **Trevor H. Drees:** data curation (equal), investigation (equal), writing – review and editing (equal). **Christoph Rosche:** data curation (equal), investigation (equal), writing – review and editing (equal). **Katriona Shea:** data curation (equal), investigation (equal), writing – review and editing (equal). **Simone Ravetto Enri:** data curation (equal), investigation (equal), writing – review and editing (equal). **Jennifer R. Gremer:** data curation (equal), investigation (equal), writing – review and editing (equal). **Balázs Deák:** data curation (equal), investigation (equal), writing – review and editing (equal). **Lauchlan Fraser:** data curation (equal), investigation (equal), writing – review and editing (equal). **Lauri Laanisto:** data curation (equal), investigation (equal), writing – review and editing (equal). **Orsolya Valkó:** data curation (equal), investigation (equal), writing – review and editing (equal). **Robert Drew Sieg:** data curation (equal), investigation (equal), writing – review and editing (equal). **Benedicte Bachelot:** data curation (equal), investigation (equal), writing – review and editing (equal). **Jennifer Firn:** data curation (equal), investigation (equal), writing – review and editing (equal).

## Funding

This work was supported by Research Ireland, 22/CC/11103, UK Research and Innovation, 22/CC/11103, Department of Agriculture, Environment and Rural Affairs, UK Government, 22/CC/11103, FP7 People: Marie‐Curie Actions, 631105 and Australian Research Council, DP120101462.

## Disclosure

Statement on inclusion: The data were collected by participants in the distributed ecology network Plantpopnet. All data collectors were invited to contribute further to the manuscript and represent a wide range of countries worldwide where the research was carried out. We provide further information in the main text of the paper on the recruitment and management of authorship for this inclusive approach, including a link to the authorship guidelines for Plantpopnet.

## Conflicts of Interest

The authors declare no conflicts of interest.

## Supporting information


**Supporting Information: S1.** The supporting materials can be found at: 1.1. Network governance principles for Plantpopnet (https://doi.org/10.6084/m9.figshare.24299050). 1.2. Authorship guidelines for Plantpopnet (https://doi.org/10.6084/m9.figshare.24299089). 1.3. Funding guidelines for Plantpopnet (https://doi.org/10.6084/m9.figshare.24299062). 1.4. Plantpopnet data Management (https://doi.org/10.6084/m9.figshare.24299116). 1.5. Abstract template for approval by steering committee (https://doi.org/10.6084/m9.figshare.24299122).
**Supporting Information: S2.** Estimated person years invested in Plantpopnet core demographic data 2014–2021. We assumed 230 working days per year. Data curation included digital data curation and physical curation of samples (seeds, leaves etc.) as well as dealing with enquiries from participants.
**Supporting Information: S3.** Recruitment of participants to the network by year of first data collection.
**Supporting Information: S4.** Timing of demographic data collected in 2022 from the ongoing Plantpopnet project and estimate of survey person days for each year of additional data. Survey person days were estimated as 3 person‐days per site per year including the field census and initial digitisation of data. Reasons for abandonment are given for 16 of the 65 sites. *Sites where the coordinator was informed. This means abandoned sites that were acknowledge by the site coordinator to the Plantpopnet data curators.
**Supporting Information: S5.** Participant survey questions (https://doi.org/10.6084/m9.figshare.24299278).
**Supporting Information: S6.** Key considerations and challenges to overcome when setting up a network with suggested solutions learned.

## Data Availability

Standard Data Product: SDP Y0.1.3 and associated summary table of Plantpopnet site characteristics provided on Dryad DOI: https://doi.org/10.5061/dryad.m37pvmdjc. An archived version of the code and raw data for the data cleaning process and production of Standard Data Product (SDP) is available on Zenodo: https://doi.org/10.5281/zenodo.16608913.
