# Supplementary material for: Quality Assurance Framework for the Design, Collection and Curation of Standard Data Products From a Distributed Ecology Network
Source: Ecol Evol. 2026 Aug 10;16(8):e74000. doi: 10.1002/ece3.74000 (PMC13456982; doi:10.1002/ece3.74000)
Supplement: Supplementary file 1 [file ECE3-16-e74000-s001.docx]

**Supporting information**

**SI** The supporting materials can be found at:

**SI 1.1**. Network governance principles for Plantpopnet (<https://doi.org/10.6084/m9.figshare.24299050>)

**SI 1.2**. Authorship guidelines for Plantpopnet (<https://doi.org/10.6084/m9.figshare.24299089>)

**SI 1.3**. Funding guidelines for Plantpopnet (<https://doi.org/10.6084/m9.figshare.24299062>)

**SI 1.4**. Plantpopnet Data Management (<https://doi.org/10.6084/m9.figshare.24299116>)

**SI 1.5**. Abstract template for approval by steering committee (<https://doi.org/10.6084/m9.figshare.24299122>)

**SI 2**. Estimated person years invested in Plantpopnet core demographic data 2014-2021. We assumed 230 working days per year. Data curation included digital data curation and physical curation of samples (seeds, leaves etc.) as well as dealing with enquiries from participants.

| **Function** | **Time estimate** | **Total person years** |
| --- | --- | --- |
| Coordination of network | 11.5 days x 7 years x 1 person | 0.35 |
| Steering Group contributions | 2.5 days x (7 years x 11 people + 3 years x 2 people) | 0.9 |
| Data collection | 738 person days (SI 4.) | 3.2 |
| Data curation | 115 days x 7 years (data curator), 12 days x 8 people (reproducible data cleaning project) | 3.9 |

**SI 3.** Recruitment of participants to the network by year of first data collection.

| Year joined | No. of new sites |
| --- | --- |
| 2014 | 3 |
| 2015 | 35 |
| 2016 | 13 |
| 2017 | 4 |
| 2018 | 3 |
| 2019 | 2 |
| 2020 | 5 |

**SI 4.** Timing of demographic data collected in 2022 from the ongoing Plantpopnet project and estimate of survey person days for each year of additional data. Survey person days were estimated as 3 person-days per site per year including the field census and initial digitisation of data. Reasons for abandonment are given for sixteen of the 65 sites. *Sites where the coordinator was informed. This means abandoned sites that were acknowledge by the site coordinator to the Plantpopnet data curators.

| Years of data | No. of sites | Survey person-days | No. of sites abandoned* | Reasons for stopping |
| --- | --- | --- | --- | --- |
| 1 | 65 | 195 | 3 | Mass mortality (1), Land owner request (1), lack of activity (1) |
| 2 | 58 | 174 | 8 | Mass mortality (2), Change in site coordinator circumstance (5), Site destroyed (land built on) (1) |
| 3 | 45 | 135 | 4 | Land owner request (1), accidental destruction (2), Change in site coordinator circumstance (1) |
| 4 | 34 | 102 | 1 | Site destroyed (land constructed on) |
| 5 | 25 | 75 | 0 |  |
| 6 | 17 | 51 | 0 |  |
| 7 | 2 | 6 | 0 |  |

**SI 5.** Participant survey questions (<https://doi.org/10.6084/m9.figshare.24299278>)
**SI 6.** Key considerations and challenges to overcome when setting up a network with suggested solutions learned.

| **Key consideration** | **Suggested solution** |
| --- | --- |
| Initial set up of a network | Define research questions and reason for large collaborative network |
| Funding | A variety of funding mechanisms/distributed funding model allows for flexibility |
| Project procedures | Having a strong project design with clear shared objectives and accessible, clear methodology and tools. Adjusted when necessary. Prioritise data collection for overall research question that every site must complete, with additional data collection through optional “add-on” studies. Choosing sites close to place of work/home for ease of sustained data collection |
| Model species selection | Longevity, spatial distribution, survivability *in situ* and *ex situ* |
| Network support | Clear roles and structures, workshops, training, and support for participants. Goals that all participants can contribute to, and having participants at all career stages and various geographic locations to solve complex questions |
| Diversity of participants | Initial steering group responsible for recruitment should consist of researchers of different career stages, genders, and geographic locations |
| Travel and carbon costs | Selection of sites close to work/home. Minimal time, material and travel |
| Data management | Consistent data collection using standard methodology i.e. having agreed upon units of measurement, file naming, column naming, and location of stored data. Detailed metadata with README file, clear steps for data processing. Development of data management plan |
| Data storage | Consistent file naming, files stored in appropriate folders, backed up on hard drives/other institute systems. Controlled access to repositories and data. |
| Use of network data and publication | Having clear protocols and processes for requesting and sharing data, submitting add-on research proposals, and authorship guidelines for publication of results using network data. Avoid publication conflicts by having steering committee approve add-ons |
| Pandemic effects | Having sites close to home/work allows for robustness, and continued data collection, to unanticipated impacts of, for example, a pandemic |
| Site coordinator instability | Changes in circumstance of site coordinator was one of the leading causes of site abandonment. Employing researchers at various career stages and involving undergrads, PhDs and post-docs in data collection at sites can encourage long-term data collection at sites |
